# Facile synthesis of boronic acid-functionalized magnetic metal–organic frameworks for selective extraction and quantification of catecholamines in rat plasma†

**DOI:** 10.1039/c8ra07356b

**Published:** 2018-12-17

**Authors:** Xinying He, Yunqiu Yu, Yan Li

**Affiliations:** Pharmaceutical Analysis Department, School of Pharmacy, Fudan University Shanghai 201203 China yanli@fudan.edu.cn yqyu@shmu.edu.cn +86-21-51980057 +86-21-51980057; Fudan University Affiliated Pudong Medical Center, Fudan University Shanghai China

## Abstract

Precise determination of the endogenous catecholamines, dopamine (DA), epinephrine (E) and norepinephrine (NE) faces substantial challenges due to their low physiological concentrations in plasma. We synthesized, for the first time, a magnetic metal–organic framework (MIL-100) composite with boronic acid-functionalized pore-walls (denoted as MG@MIL-100-B composite) using a metal–ligand-fragment coassembly (MLFC) strategy. The composites were then applied as an effective magnetic solid-phase extraction (SPE) sorbent for determination of trace catecholamine concentrations in rat plasma through coupling with HPLC-MS/MS. The obtained nano-composites exhibited high magnetic responsivity, uniform mesopores, large specific surface area, and boronic acid-functionalized inner pore-walls. Catecholamines in rat plasma were extracted through interaction between the *cis*-diol structures and the boronic acid groups in the MG@MIL-100-B composites. Extraction conditions were optimized by studying SPE parameters including adsorption and desorption time, elution solvent type, pH conditions and adsorbent amount. With our approach, the detection limits (S/N = 3) were as low as 0.005 ng mL^−1^ for DA and E, and 0.02 ng mL^−1^ for NE. Intra- and inter-day precision ranged from 2.84–6.63% (*n* = 6) and 5.70–11.44% (*n* = 6), respectively. Recoveries from spiking experiments also showed satisfactory results of 94.40–109.51%. Finally, the MG@MIL-100-B composites were applied successfully to determine catecholamine concentrations in rat plasma.

## Abstract 2

MG@MIL-100-B takes magnetic Fe_3_O_4_ microparticles as core and coated by MIL-100-B shows great potential as a SPE absorbent material for the analysis of catecholamines, due to its rapid magnetic separation, outstanding sensitivity and selectivity.

## Graphical abstract

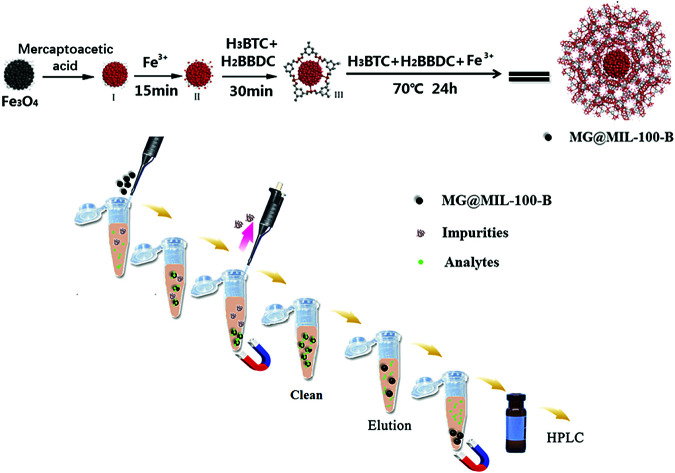

## Introduction

Catecholamines, *i.e.* epinephrine (‘E’, also known as adrenaline), norepinephrine (‘NE’, also known as noradrenaline) and dopamine (‘D’), act as hormones or neurotransmitters peripherally and centrally.^1^ Catecholamines also serve as biomarkers for the diagnosis and subsequent management (assessment of prognosis, and effectiveness of treatment) of neuroendocrine and cardiovascular diseases as they participate in a variety of regulatory systems and metabolic processes.^1–3^ Inadequate dopamine neurotransmission is associated with Parkinson's disease and schizophrenia.^4^ Catecholamine synthesizing tumors such as neuroblastoma, paraganglioma or pheochromocytoma can be life-threatening as they cause remarkable increases in catecholamine concentrations in plasma as well as urine.^3^ Given this, it is important to be able to accurately quantitate concentrations of endogenous catecholamine in biological samples.^4^

Various methods have been developed to detect catecholamines in biological samples. Traditional analytical approaches involve high-performance liquid chromatography (HPLC) coupled with electrochemical,^5^ fluorimetric,^6^ chemiluminescence,^7^ ultraviolet or mass spectrometry (MS) detection.^8^ However, these approaches are typically time-consuming, and are not sufficiently sensitive to quantify endogenous catecholamines. The application of liquid chromatography-tandem mass spectrometry (LC-MS/MS) in quantifying catecholamine concentrations has been progressively more common recently. This is due to the high selectivity and sensitivity of this method.^9^

Catecholamine assay in biological samples is difficult because of their presence in extremely low concentrations, their chemically instability and their vulnerability to interference from other compounds. Therefore, selective extraction of catecholamines prior to LC-MS/MS analysis is an indispensable step. To address this challenge, many manuals and semi-automated sample clean-up strategies have been proposed.^10^ Traditional techniques generally need complex sample preparation procedures. These include precipitation, distillation and liquid–liquid extraction, which are not only time-consuming but also prone to sample loss. Solid-phase extraction (SPE) methods including C18, C30, alumina or HLB SPE sorbents have also been reported to effectively extract and enrich of catecholamines from biological samples such as serum, plasma and urine.^11^ However, these approaches still suffer from interferences by matrix constituents especially large molecules like proteins.^12^ This indicates that it is important to design and create new SPE sorbents for specific capture of catecholamine.

Metal–organic frameworks (MOFs) are a new advanced hybrid crystal material consisting of metal ions and organic ligands.^13^ MOFs are customizable, porous, photometric, structurally flexible, and possess excellent high bearing capacity characteristics. This range of properties has resulted in MOFs having applications in a range of different fields, some excel in chemical detection and separation,^14^ others are high efficient catalysis,^15^ and still others have good applications in sensing and biomedical therapy.^16^ Recently, the combination of MOFs and magnetic nanoparticles has been demonstrated to have potential as a new type of SPE adsorbent. For example, Sun *et al.* described a magnetic ZIF-67 for selective enrichment of glycans.^17^ Yang *et al.* fabricated Fe_3_O_4_@MIL-100 magnetic microspheres to remove Cr(vi) from aqueous solution.

Recently, boronate affinity chromatography has become a research area of considerable interest.^18^ The high affinity of boronic acid molecules towards biomolecules, such as catecholamines, that contain a *cis*-diol structure means that, in mild alkali or neutral aqueous solutions, stable cyclic esters may be formed with *cis*-diol moieties and boronic acid.^19,20^ These characteristics suggest that magnetic MOFs and boronate-functionalized materials may have a potential application as SPE absorbents for selective enrichment and extraction of catecholamines from complex systems.^21^ Nevertheless, little research has focused on this area. There are several possible reasons for this. First, only a few suitable ligands contain boronic acid groups that can be used for functionalized MOFs. Second, the accessional functional groups fill more space, which significantly reduces the enrichment capacity of the resultant MOFs because of the declining molecule-accessible surface area and pore volumes available for reactions to take place. Third, synthesis of a combination of magnetic nanoparticles and MOFs often involves a tedious and complicated stepwise reaction strategy.^22^ Finally, most constructed MOFs are unstable in matrix solutions.^23^ Thus, there is a need to develop a new generation of magnetic MOFs with integrated boronic acid functionality that possess satisfactory enrichment capacity and sufficient chemical stability for catecholamine enrichment analysis. Therefore, the aim of our research was to develop and operate a simpler and faster one-step method for the synthesis of a boronic acid modified magnetic MOF absorbent.

To achieve this goal, we adopted the metal–ligand-fragment coassembly (MLFC) strategy for functional group modification. As compared with the conventional pre- and post-functionalization method, the MLFC strategy was used to attach boronic acid functional groups to the MOFs *in situ* rather than achieving this through a decrease in pore volume. We also successfully used Fe_3_O_4_ precursor as a crystal seed to grow boronic acid functionalized MOFs in the matrix solution step. MIL-100(Fe) (MIL: Materials of Institut Lavoisier) was certified to be a stable MOF material in matrix solution, so we chose to use it as a basic skeleton adopting FeCl_3_ to introduce Fe^3+^ as a metal center and 1,3,5-benzene tricarboxylic acid as a primary organic ligand. MIL-100-B (boronic acid functionalized MIL-100) was prepared with active boronic acid suspended in the MIL-100 cavity by employing commercially available 5-boronobenzene-1,3-dicarboxylic acid as a ligand fragment to introduce the functional component. This was a simpler way of obtaining core–shell composites compared with the traditional step-by-step method. We then took advantage of the potent affinity of boronic acid to *cis*-diol containing biomolecules (catecholamines),^5,24^ with the MG@MIL-100-B nanoparticles further employed to trap catecholamines from biological samples. The magnetic porous structure and numerous boronic acid functional groups made the MG@MIL-100-B nanoparticles separate rapidly from the sample matrix, excluding the high molecular weight protein with high concentration and be excellent selectivity.

To our knowledge, this is the first synthesis method for MG@MIL-100-B, with the enrichment efficiency further investigated using trace analysis of catecholamines in a biological matrix. This research sought to explore the feasibility of using MG@MIL-100-B composites for selective extraction of catecholamines, and to develop a sensitive and high-efficiency analysis method for catecholamines in biological matrices through incorporation of an LC-MS/MS method.

## Results and discussion

### Characterization of MG@MIL-100-B composites

The morphology of the MG@MIL-100-B composites was identified by TEM. The TEM images revealed that MG@MIL-100-B composites have a core–shell structure with a mean diameter of about 250 nm, and MOF layer thickness of about 50 nm (Fig. 2). Fig. 2a shows a typical TEM image of a magnetic Fe_3_O_4_ nanosphere. Fig. 2b and c show a typical MOF structure (thickness of 40–50 nm) indicating successful coating of MIL-100-B on the whole Fe_3_O_4_ nanosphere.

**Fig. 1:**
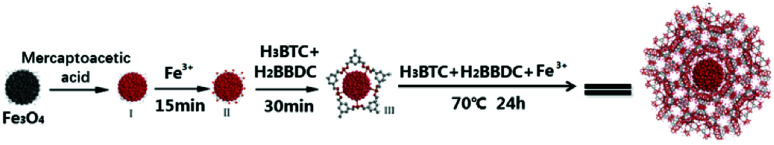
The synthetic protocol of MG@MIL-100-B composites.

**Fig. 2:**
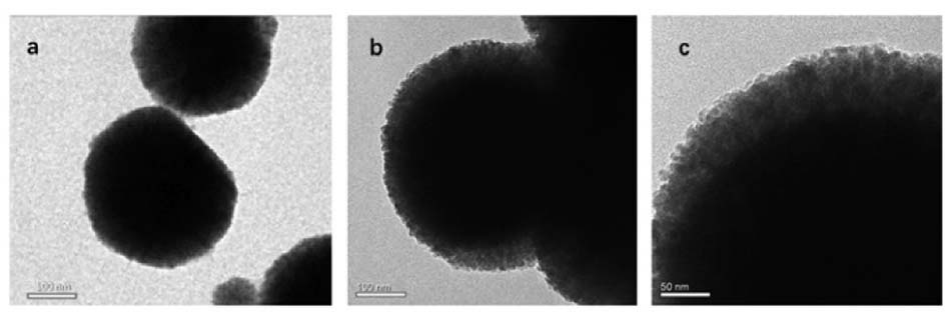
TEM images of (a) Fe_3_O_4_ particle, (b and c) the MG@MIL-100-B composites.

**Fig. 3:**
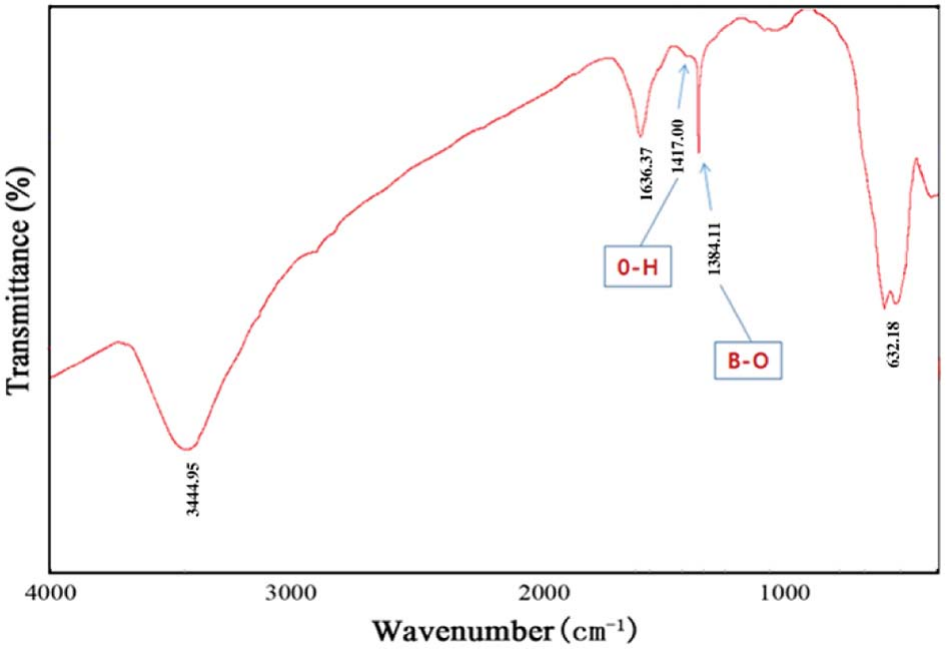
FT-IR spectra were recorded to characterize MG@MIL-100-B nanoparticles of the MG@MIL-100-B composites.

FT-IR spectra were recorded to characterize MG@MIL-100-B nanoparticles as shown in Fig. 3. A strong adsorption peak appears at 587 cm^−1^, which could be attributed to Fe–O–Fe vibration. Two absorption peaks at 1385 and 1417 cm^−1^ are observed, which can be ascribed to B–O stretching vibration and O–H bending vibration, respectively. The sharp bands at 1636 cm^−1^ correspond to the asymmetric and symmetric vibrations of –COO^−^ groups. These typical adsorption peaks of groups clearly confirmed the successful self-assembly of Fe_3_O_4_ microspheres and MIL-100-B.

**Fig. 4:**
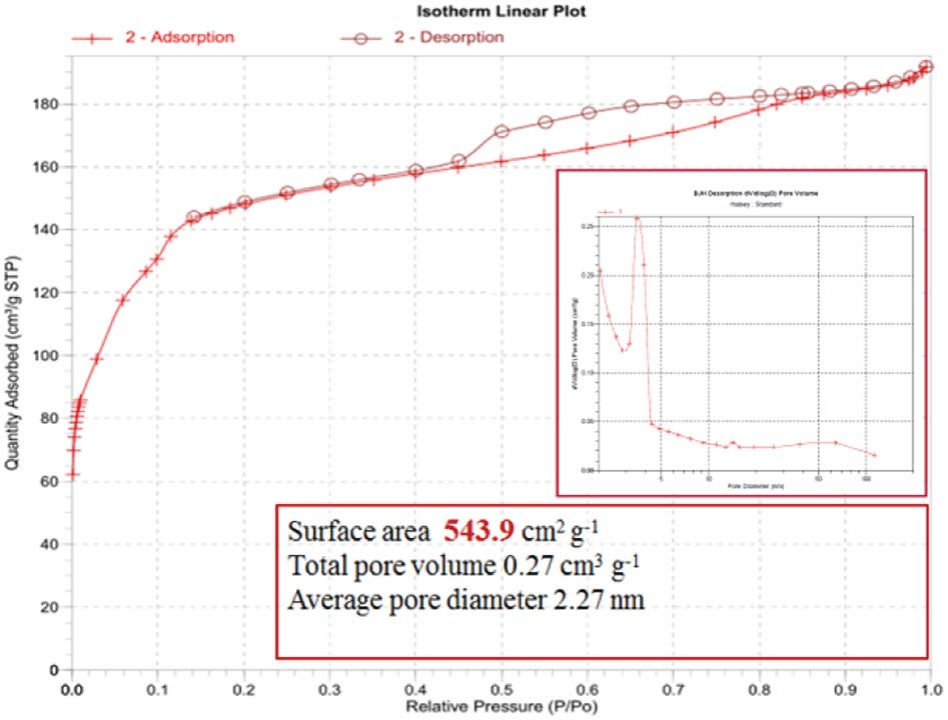
Nitrogen adsorption–desorption isotherms and pore size distribution (inset) of the MG@MIL-100-B composites (recorded at 200 °C).

**Fig. 5:**
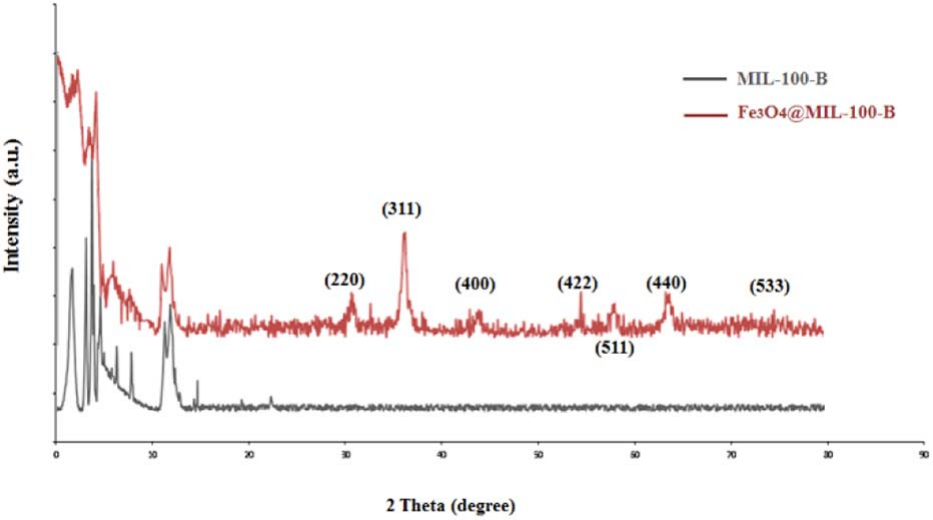
Wide-angle XRD spectrum of MIL-100-B and MG@MIL-100-B composites.

To study the porous structure of MG@MIL-100-B nanoparticles, N_2_ adsorption–desorption isotherms were recorded at 200 °C in accordance with reported data for other MOF materials. Fig. 4 shows the nitrogen adsorption–desorption isotherms of MG@MIL-100-B composites to have a representative IV-type curve for the porous material with a unique H4 hysteresis loop. An obvious capillary condensation step emerged between 0.4 and 0.6 *P*/*P*_0_ also revealing that the material had a mesoporous structure. A uniform pore size of 2.27 nm was calculated using the Barrett–Joyner–Halenda method, which was consistent with the pore size distribution curve (Fig. 4, inset). Total pore volume of the composites was 0.27 cm^3^ g^−1^ and BET surface area was 543.9 cm^2^ g^−1^. The values are lower than the other MOF materials previously reported because of the presence of Fe_3_O_4_ core. The nitrogen adsorption–desorption isotherms of Fe_3_O_4_ microsphere (in Fig. S3†) showed that the total pore volume of the microsphere was 0.028 cm^3^ g^−1^ and the BET surface area was 27.5 cm^2^ g^−1^. These values were much lower than that of the MG@MIL-100-B materials which proved the successful synthesis of the pore structure, MIL-100-B, on the surface of the Fe_3_O_4_ microsphere. In the recorded wide-angle XRD spectrum (Fig. 5) of MG@MIL-100-B composites and MIL-100-B, a group of diffraction peaks at around 30.19°, 35.44°, 43.29°, 53.69°, 57.13° and 62.91° were indexed to the (220), (311), (400), (422), (511) and (440) planes, respectively. These related to the Fe_3_O_4_ nanospheres in the composites. Compared with the XRD pattern of MIL-100-B, it can be seen that MIL-100-B were also synthesized on the magnetic Fe_3_O_4_ microspheres.

### Optimization of extraction conditions

Many factors influenced extraction conditions. Adsorbent amount (2, 4, 8, 10, 15 and 20 mg), elution solvent type (acetonitrile, methanol, methanol/water (1 : 1 v/v), 1% formic acid methanol, 5% formic acid methanol, and 5% acetic acid methanol), adsorption time, desorption time (from 2 to 12 min) and adsorption pH (5.0, 6.0, 7.0, 8.0, 8.5 and 9.0) were all evaluated systematically to optimize extraction performance.

### Optimization of the amount of MG@MIL-100-B composites

**Fig. 6:**
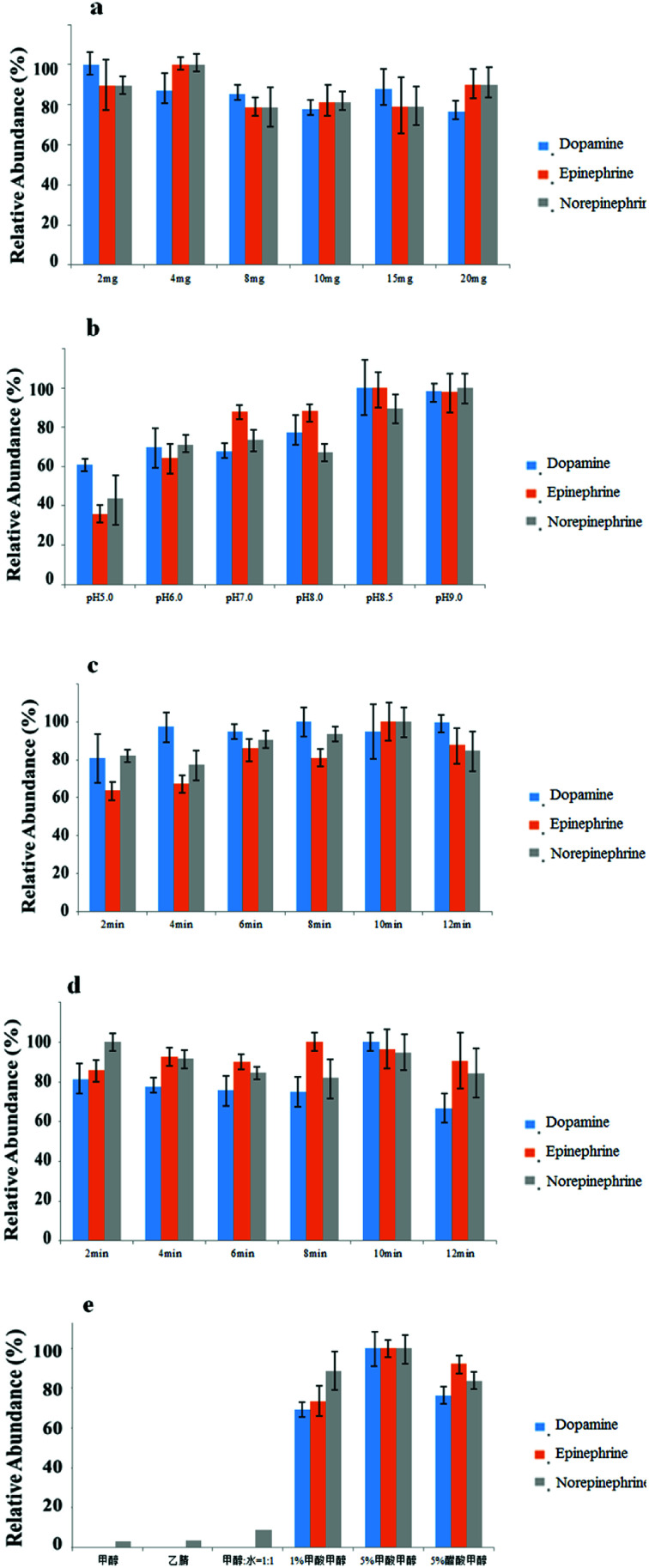
Effect on SPE performance of (a) different amounts of the MG@MIL-100-B composites; (b) pH of the matrix; (c) different adsorption times; (d) different desorption times. (e) Different elution solvent types; error bars represent 3 separate treatments.

In the experiment, a methanol suspension of the composites was used instead of adding MG@MIL-100-B dry powder directly. The amount of composites was thus precisely controlled through introducing various volumes of the suspension and following supernatant removal. This step was the premise for μSPE adsorbents. The amount of MG@MIL-100-B adsorbents was considered to be the most crucial factor influencing extraction efficiency. For optimal extraction, it is essential to have sufficient adsorbents because the adsorbents have finite capacity to carry the target analytes. In this section, we optimized use of MG@MIL-100-B composites by varying the amount from 2 to 20 mg (2, 4, 8, 10, 15 and 20 mg). The other SPE conditions were fixed as 10 min for desorption time, 10 min for adsorption time, 400 μL of 5% formic acid methanol solution as elution solvent, and a pH of 8.5. Fig. 6a, shows that extraction was optimally efficient at less than 4 mg of the MG@MIL-100-B composites and there was no remarkable improvement when more than 4 mg was added. Hence, 4 mg was chosen as the optimal MG@MIL-100-B composite amount.

### Optimization of matrix pH of matrix

In catecholamine binding experiments, pH is the most important parameter affecting the reversible binding procedure. This is because the affinity of catecholamines for MG@MIL-100-B composites is based on the typical boronate esterification reaction. In this reaction, the boronic acid group reacts with the *cis*-diol catecholamine moiety to form a cyclic ester in an alkaline aqueous medium, and these esters dissociate when the solution pH changes from neutral to acidic.^24^ In this work, different pH values (5.0, 6.0, 7.0, 8.0, 8.5 and 9.0) of the sample matrix were studied and the other SPE conditions were fixed as 4 mg of MG@MIL-100-B composites, 10 min of adsorption time, 400 μL of 5% formic acid methanol solution as elution solvent and 10 min of desorption time. Fig. 6b shows that the highest extraction efficiency was obtained when the matrix was at a pH of around 8.5. We deduced that the low pH value enables the highest boronate esterification reaction efficiency whereas high pH values compromise stability of the analytes and materials. Therefore, the optimal pH value of the sample matrix was chosen to be 8.5 for use in all subsequent experiments.

### Optimization of adsorption and desorption time

In this part of the study, the effect of adsorption time on binding capacity and desorption time on releasing capacity were investigated. We studied adsorption and desorption times of 2 to 12 min and kept the other SPE conditions stable at pH 8.5, 400 μL of 5% formic acid methanol solution as elution solvent, and 4 mg of MG@MIL-100-B composites. Accordingly, adsorption or desorption times were controlled at 10 min when the other variables changed. Fig. 6c and d show that the highest extraction efficiency was obtained when the adsorption and desorption times were both controlled at 10 min. This was long enough for the adsorption and elution of analytes with MG@MIL-100-B. However, a longer time could achieve the same efficiency and even ensure a more complete reaction, but may damage the structure of the materials and the stability of the analytes. Therefore, the optimal adsorption and desorption time was chosen as 10 min.

### Optimization of the elution solvent type

Elution solvent type is another factor that significantly affects extraction efficiency. Use of an SPE method prior to HPLC analysis facilitates obtaining reliable and reproducible analytical results. In this section, the other SPE conditions were fixed as 4 mg of MG@MIL-100-B composites, 10 min adsorption and desorption times, and 400 μL of elution solvent. The six kinds of commonly used elution solvents tested were acetonitrile, methanol, methanol/water (1 : 1 v/v), 1% formic acid methanol, 5% formic acid methanol and 5% acetic acid methanol. Fig. 6e suggests that 5% formic acid methanol solution had better eluting ability than the five other solvents, which was also in accordance with some literature.^19^ One interpretation may be that formic acid, with the strongest acidity, has the ability to promote the dissociation reaction to completion.

Collectively these findings indicated that the optimal extraction conditions were pH 8.5 of the sample matrix, 4 mg of MG@MIL-100-B composites, 10 min for both adsorption and desorption times, and a single elution with 400 μL of 5% formic acid methanol solution.

### Method validation

Tables 2 and 3 list the criteria for validating the proposed method. The linearity (*R*^2^ > 0.9910 for all) ranged from 0.1–8 ng mL^−1^ for NE and from 0.01–2 ng mL^−1^ for E and DA in the case of the conditions optimized. The limits of quantification (LOQ: S/N = 10) and detection (LOD: S/N = 3) were 0.1 and 0.02 ng mL^−1^ for NE, and 0.01 and 0.005 ng mL^−1^ for E and DA, respectively.

**Table 1:** LC-MS/MS parameters for DA, E, NE, and DHBA (I.S.)

Analyte	Relative retention time	ESI (+/−)	Parent ions (*m*/*z*)	Daughter ions (*m*/*z*) SRM_1_/SRM_2_	DP(V)	CE (eV)	EP(V)	CXP(V)
Dopamine (DA)	4.85 ± 0.02	(+)	154.1	137.0^a^/91.0	41	13/33	10	10
Epinephrine (E)	3.32 ± 0.02	(+)	184.1	166.1^a^/107	45	14/30	10	10
Norepinephrine (NE)	2.80 ± 0.02	(+)	170.1	152.0^a^/107.2	38	12/29	10	10
3,4-Dihydroxybenzylamine (DHBA, I.S.)	3.76 ± 0.02	(+)	140.1	123.0^a^/77.0	33	14/34	10	10

a The product ion used for quantification.

**Table 2:** Linearity, LOD and LOQ of the proposed method, reproducibility of the MG@MIL-100-B composites^a^

Analyte	LOD (ng mL^−1^)	LOQ (ng mL^−1^)	Linear range (ng mL^−1^)	Determination coefficient (*R*^2^)	Liner equations	Reproducibility of the MG@MIL-100-B composites (inter-batch precision, RSD (%))
DA	0.005	0.01	0.01–2	0.9943	*y* = 148.47*x* + 4.3545	4.89
E	0.005	0.01	0.01–2	0.9911	*y* = 638.86*x* + 52.208	5.29
NE	0.02	0.1	0.10–8	0.9909	*y* = 17.039*x* + 5.6067	5.87

a Inter-batch precision: *n* = 6.

**Table 3:** Extraction yields, recoveries, intra-day and inter-day precisions of the proposed method^a^

Analyte	Fortified concentration (ng mL^−1^)	Matrix effect (%)	Recovery (%)	Intra-day precision RSD (%)	Inter-day precision RSD (%)
DA	0.05 (low)	86.71	102.93	6.63	9.16
	0.5 (medium)	93.30	99.46	5.87	8.63
	2 (high)	95.70	100.56	2.84	6.37
E	0.05 (low)	94.12	100.34	6.55	11.44
	0.5 (medium)	89.31	100.53	5.31	8.44
	2 (high)	115.22	101.49	4.71	7.39
NE	0.05 (low)	90.46	101.31	5.87	10.47
	0.5 (medium)	96.53	101.16	3.83	5.70
	2 (high)	115.82	99.35	4.03	9.37

a Intra- and inter-day precisions: *n* = 6.

Precision was ascertained by the inter- and intra-assay RSDs of each three different concentration level samples. All the RSDs were less than 11%, which indicated good accuracy and reproducibility. Acceptable recoveries were in the range of 94.40–109.51%. Matrix effect of three analytes was ranging from 86.71% to 115.82%, which is well within acceptable ranges (<20%), suggesting no evident matrix effect in the study of samples treated.

To assess recyclability, we cleaned MG@MIL-100-B composites with deionized water and methanol at least ten times after initial use. Comparison of the recycled composite with the newly synthesized one indicated no significant difference in reaction progress or extraction efficiency. Moreover, the MG@MIL-100-B composites still possessed a perfect extraction performance after being used and recycled eight times.

### Comparison with other methods

The acquired results were compared with those obtained by typical conventional SPE methods (Oasis HLB, C18, C30, and also Oasis HLB after C18) or other sample preparation methods (liquid-phase extraction, aluminum oxide extraction and dilution) to determine the advantages of the method proposed here. Table 4 indicates that our method produces similar or improved quantitation results compared with the other methods. Further, our sample preparation procedure was simpler and timesaving.

**Table 4:** Comparison of the proposed method with other reported methods for catecholamines analysis in plasma sample

Ref.	Sample preparation procedure	Analytical method	Sample pretreatment time (min)	Analyte	LOD	LOQ	Linear range (ng mL^−1^)	Recovery (%)	Precision, RSD (%)
					(ng mL^−1^)	(ng mL^−1^)			
This work	μSPE with MG@MIL-100-B	LC-MS/MS	∼25	DA	0.005	0.01	0.01–2	99.46–102.93	2.84–6.63
				E	0.005	0.01	0.01–2	100.34–101.49	4.71–6.55
				NE	0.02	0.1	0.1–8	99.35–101.31	3.83–5.86
3	Tomtec Quadra 96 liquid handing robot to expedite aluminum oxide extraction	LC-MS/MS	120	DA	—	2.5	2.5–500	66.70–67.80	1.45–10.90
28	Liquid-phase extraction with alumina method	LC-amperometry	50	DA	—	0.31	3.06–306.4	70–100	<20
				E	—	0.37	3.66–366.4		
				NE	—	0.34	3.38–338.4		
	Liquid-phase extraction with molecular mass cut-off method	LC-coularray	60	DA	—	0.031	3.06–306.4	70–100	<5
				E	—	0.037	3.66–366.4		
				NE	—	0.034	3.38–338.4		
29	Hexane wash – centrifugation – nitrogen dryness – redissolve	LC-ECD	>60	NE	0.6	2	20–60	—	2.88–4.35
18	Dilute with the sample dilution buffer	LC-peroxyoxalate chemiluminescence	—	DA	—	0.06	—	—	—
				E	—	0.02	—	—	—
				NE	—	0.03	—	—	—
30	SPE [Oasis HLB/Isolute MFC18/C30 cartridges]	LC-amperometric	>30	DA	0.01	0.04	0.01–2	>85%	<10%
				E	0.01	0.02	0.01–2		
				NE	0.04	0.1	0.04–5		
31	SPE [Oasis HLB]	LC-coulometric	>30	DA	0.012	0.04	0.04–1.6	97	3.9
				E	0.012	0.04	0.04–1.6	93	3.8
				NE	0.012	0.04	0.04–1.6	96	4.3
32	SPE [Oasis HLB followed by C18]	LC-amperometric	>30	DA	0.1	0.2	0.2–10	80	3.2–5.9
				E	0.1	0.2	0.2–10	90	2.9–4.8
				NE	0.15	0.2	0.2–10	90	1.2–5.6

Table 4 also briefly compares our approach against published methods for extracting catecholamines from plasma samples. The comparison proves that using MG@MIL-100-B composites as a sorbent for trace analysis of catecholamines in plasma is convenient, sensitive and rapid. The magnetic Fe_3_O_4_ microsphere simplified the sample pretreatment process, MIL-100-B improved the enrichment ability and purified the sample matrix, and the LC-MS/MS system gave high analyte selectivity and sensitivity. Our sample pretreatment strategy promises to provide an alternative for the selective enrichment of small molecules in a complex matrix.

### Application to the analysis of real plasma samples

MG@MIL-100-B composites were used as SPE sorbents and combined with HPLC-MS/MS to detect catecholamines in rat plasma after validating the proposed method. Six anesthetized male SD rats (8–10 weeks old, 300–400 g body weight), were provided by the mouse supply center of the School of Pharmacy at Fudan University. Blood was drawn from both orbital veins, and the plasma fraction extracted after centrifuging (3000*g*) the blood at 4 °C for 20 min. All animal procedures were performed in accordance with the Guidelines for Care and Use of Laboratory Animals of Fudan University and experiments were approved by the Animal Ethics Committee of Fudan University School of Pharmacy.

**Fig. 7:**
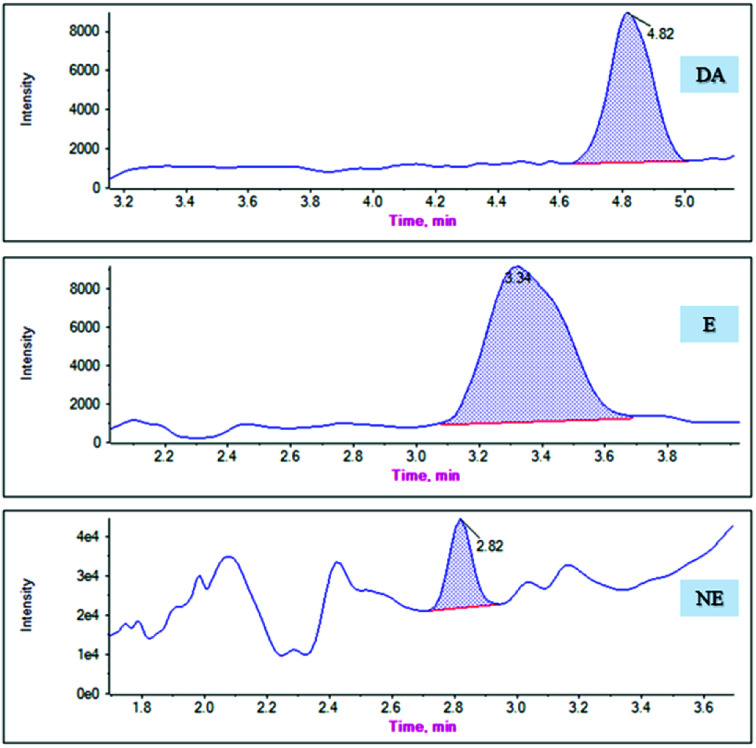
The chromatograms of DA, E and NE in actual plasma samples.

The mean ± standard deviation catecholamine concentrations in the six rat plasma samples were calculated as 0.75 ± 0.32 ng mL^−1^ (DA), 0.90 ± 0.23 ng mL^−1^ (E), and 1.50 ± 0.22 ng mL^−1^ (NE). Fig. 7 presents the chromatograms of the actual plasma samples. The analytical results of these samples revealed that the measured endogenous concentrations was in the linear range of our method, which is applicable to use in a clinical study.

## Experimental

### Chemicals and reagents

1,3,5-Benzene tricarboxylic acid (BTC) and 5-boronobenzene-1,3-dicarboxylic acid (BBDC) were purchased from Aladdin chemical reagent Corporation (Shanghai, China, http://www.aladdin-e.com/). Ethylene glycol, ferric trichloride hexahydrate, sodium acetate, formic acid, trisodium citrate, sodium hydroxide, ethanol, ammonium formate, ethylenediaminetetraacetic acid disodium salt dihydrate (EDTA·2Na) and mercaptoacetic acid (MAA) were all of analytical grade and purchased from Sinopharm Chemical Reagents Co. Ltd (Shanghai, China, http://www.sinoreagent.com/). All of the catecholamines, including dopamine hydrochloride (DA), epinephrine (E), norepinephrine (NE) and internal standard 3,4-dihydroxybenzylamine (DHBA) were purchased from Sigma-Aldrich (St. Louis, MO, https://www.sigmaaldrich.com/). The chemical structures of the analytes are presented in Fig. S1.† Methanol and acetonitrile were of HPLC grade (99.9%) and were purchased from Merck (Darmstadt, Germany, http://www.merck-china.com/). Phosphate-buffered saline (PBS) was obtained from Dalian Meilun Biotechnology Company. Deionized water used in the whole experiment was prepared by a Milli-Q system (Millipore, Bedford, MA, http://www.millipore.bioon.com.cn/). Rat plasma was purchased from Nanjing SenBeiJia Biological Technology Corporation (Nanjing, China, http://njsbjsw.bioon.com.cn/).

Standard stock solutions of DA, E, NE and DHBA (I.S.) were prepared by dissolving each compound in 0.1% hydrochloric acid solution (1 g L^−1^ Na_2_S_2_O_5_ as antioxidant) to reach a concentration of 1 mg mL^−1^. Stock solutions were stored at −20 °C in dark and were proven to be stable under storage conditions. Working standard solutions were prepared by diluting the stock solutions with 0.1% hydrochloric acid solution before use. Rat plasma were stored in polypropylene tubes at −20 °C in dark to avoid decomposition.

### Apparatus

Transmission electron microscopy (TEM) images were taken with a JEOL 2011 microscope (Japan) at 200 kV. Fourier transform infrared (FT-IR) spectra were collected on a Nicolet Nexus 470 Fourier spectrophotometer (USA) using KBr pellets. The nitrogen sorption isotherms, pore size distribution and Brunauer–Emmett–Teller (BET) surface area were measured at 250 °C with a Micromeritics TriStar 3000 analyzer (USA). Powder X-ray diffraction (XRD) measurements were performed with a Bruker D4 X-ray diffractometer with Ni-filtered Cu Kα radiation (40 kV, 40 mA). For all of the chemicals and reagents, an AB204-N analytical balance (Mettler Toledo, Switzerland) was used for weighing. Stirring was performed by an HD2004W constant speed mechanical stirrer (Sile, China).

### LC-MS/MS conditions

Chromatographic separation was achieved using a UHPLC system consisting of an Agilent Series 1290 degasser (Agilent Technologies, Waldbronn, Germany), an autosampler (Model no. G4278A) with a thermostat (Model no. G4270), a binary pump (Model AU7 no. G4220A), and a thermostated column department (Model no. G1316A) equipped with an inspire PFP analytical column (150 × 4.6 mm, 5 μm particle size; DIKMA, USA) at a flow rate of 800 μL min^−1^. The mobile phase was a mixture of (A) 0.1% formic acid aqueous solution and (B) methanol, both of which were filtered through 0.22 μm membrane filters before use. Different chromatographic conditions were tested and a gradient elution program was finally chosen. A total gradient program of 8 min was applied as follows: 0–1 min, 10% B; 1–2 min, 10–20% B; 2–5 min, 20% B; 5–5.5 min, 20–10% B; 5.5–8 min, 10% B. The total run duration was 5.5 min (exclude the equilibration step) with the flow rate maintained at 0.8 mL min^−1^ throughout the entire run. The column temperature was held at 30 °C. The injection volume was 5 μL.

The MS was set in a positive electrospray ionization (ESI) mode on a 6500 triple quadrupole MS/MS (AB SCIEX, Framingham, MA). Analyst 1.6.2 and MultiQuant 3.0 software (ABSCIEX) were used for data acquisition and processing. Nitrogen was the drying and collision gas. Catecholamine analytes and the I.S. were dissolved in the mobile phase to 100 μg mL^−1^ and infused individually into the mass spectrometer to optimize the MS/MS parameters. Electrospray positive ionization (ESI+) and multiple reactions monitoring (MRM) mode were used. For each compound, two ion transitions were set in order to reach the highest selectivity and sensitivity. Typical source parameters were as follows in positive-ionization mode: ion spray voltage of 5.5 kV, curtain gas at 30 psi, nebulizer (gas 1) and turbo gas (gas 2) at 50 psi, and turbo gas temperature of 450 °C. Table 1 listed the individual MS/MS parameters of target analytes and the I.S.

### Synthesis of MG@MIL-100-B composites

Fig. 1 presents the synthetic protocol of composites of MG@MIL-100-B. Fe_3_O_4_ nanoparticles were fabricated by performing a solvothermal reaction following the report done before.^25^ Through TEM (short for transmission electron microscope), the size and morphology of magnetic particles can be clearly observed.

Afterwards, the MG@MIL-100-B composites were fabricated by a strategy of MLFC (short for metal–ligand-fragment coassembly) for introducing boronic acid functionalized groups into their cavities.^26^ The specific steps are as follows: 350 mg of the above Fe_3_O_4_ nanoparticles were washed 3 times with ethanol, and 75 mL of ethanol containing 0.58 mm MAA was added. Then the mixture was gently stirred for 24 hours as protected by nitrogen. The resulting product (expressed as Fe_3_O_4_-MAA) was collected with a magnet and cleaned several times with ethanol until no pungent odor was found. Then the product was dried for 12 h at 60 °C in the case of vacuum. After above steps, MAA-functionalized Fe_3_O_4_ was obtained.^27^

Dispersed 0.10 g of the MAA-functionalized Fe_3_O_4_ particles in 5 mL ethanol solution of FeCl_3_·6H_2_O (0.027 g) with subsequent heating at 70 °C for 15 min, and then were separated with a magnet and washed with ethanol thrice. Introduced the samples to ethanol solution of 0.0126 g BTC and 0.0085 g BBDC (BTC/BBDC feed ratio = 1.5 : 1) with subsequent heating at 70 °C for 30 min, and then were separated with a magnet and washed with ethanol thrice. Thus, this work acquired the Fe_3_O_4_@MIL-100-B precursors. By stirring, dispersed Fe_3_O_4_@MIL-100-B precursors in the FeCl_3_·6H_2_O (0.270 g), BTC (0.1260 g) and BBDC (0.0851 g) in ethanol mixed solution (60 mL). Placed the mixture in a 200 mL PTFE-reactor and heated at 70 °C for 24 h and then decreased the temperature to ambient temperature. Eventually, the samples were separated with a magnet and washed with ethanol thrice. Afterwards, dried the samples under vacuum at 60 °C for 12 h. Thus, the MG@MIL-100-B composites were obtained.

### Rat plasma samples

Rat plasma was maintained at 37 °C for 48 h to oxidatively decrease endogenous NE, E and DA to concentrations below the limit of detection.^18^ Matrix stabilizer solution (2 mL of 50 mg mL^−1^ sodium metabisulfite in 0.1% hydrochloric acid solution) was added to 98 mL of the post-degradation rat plasma with homogenous mixing. The diluted, stabilized and heat-treated rat plasma served as an artificial blank (surrogate) matrix to prepare standards for the calibration curve in concentration ranges of 100.0–8000 pg mL^−1^ for NE, and 10.00–2000 pg mL^−1^ DA and E. The plasma samples of rats in the control group were similar to those of the control group, and the samples were analyzed by matrix stabilizer solution (49 : 1). The internal standard, DHBA, was prepared in a 100 ng mL^−1^ concentration.

### Optimization of extraction procedure parameters

The extraction efficiency of catecholamines from rat plasma was determined by analyzing quality control samples. SPE conditions were optimized by applying the single-factor test to obtain high extraction efficiency. Parameters including adsorption time, elution solvent type, adsorbent amount, desorption time and adsorption pH conditions were studied systematically. Catecholamine standard solution was added to blank rat plasma samples and mixed into a solution containing 1 ng mL^−1^ DA, E and NE, and 100 ng mL^−1^ DHBA. Fig. S2† shows the systematic extraction procedure. MG@MIL-100-B composite suspension (20 mg mL^−1^ in methanol) was introduced to PPE tubes by subsequently isolating MG@MIL-100-B composites and removing supernatant under magnetic field. Six adsorbent levels (2, 4, 8, 10, 15 and 20 mg) were analyzed using various suspension volumes. Plasma matrix (1 mL) of varying pH values (5.0, 6.0, 7.0, 8.0, 8.5 and 9.0, adjusted using ammonia) was introduced into the PPE tubes and mixed using SPE adsorbents by vortex for variable durations (2, 4, 6, 8, 10, and 12 min). An external magnetic field was then employed to isolate the MG@MIL-100-B composites bound to target catecholamines. Subsequently, MG@MIL-100-B composites were rinsed with 500 μL of 5% formic acid methanol solution. Certain volumes of the elution solvent (acetonitrile, methanol, methanol/water (1 : 1 v/v), 5% acetic acid methanol, 5% formic acid methanol and 1% formic acid methanol) were employed to elute catecholamines. After vortex vibration treatment for a specific desorption time (2, 4, 6, 8, 10 and 12 min), the supernatant (eluate that contained the target analytes) was collected under magnetic field while the MG@MIL-100-B composites were isolated. Eventually, the eluate was filtered and injected into the HPLC-MS/MS system for analysis.

### Method validations

By analyzing standard fortified blank plasma samples under optimized experimental conditions, the method was validated for extraction efficiency, matrix effect, specificity, linear range, sensitivity, precision and accuracy. Linearity of the analytical method was performed using seven concentrations of DA and E (0.01, 0.02, 0.05, 0.1, 0.5, 1 and 2 ng mL^−1^), and NE (0.1, 0.4, 0.8, 2, 4, 6 and 8 ng mL^−1^). The method for calculating the peak area ratio was to divide the area of each analyte by the standard DHBA. Linearity was evaluated using a weighted (1/*x*^2^) least-squares linear regression analysis.

Accuracy and precision (inter- and intra-run) were calculated under three concentrations of quality control samples for DA and E (0.05, 0.5 and 2 ng mL^−1^), and NE (0.4, 2 and 6 ng mL^−1^). Six replicates of each quality control concentration were analysed to ascertain the accuracy and precision within one day. This process was repeated three times over more than three days to ascertain the accuracy and precision between days. Recoveries were calculated as measured content *versus* fortification concentration. The lower limits of quantification and detection (LOQ and LOD, respectively) were determined using 5 μL of the standard mixtures (10 and 3 signal/noise ratios, respectively). Since MG@MIL-100-B composites have limited capacity to carry target molecules, when treating samples with high analyte concentration, we used increased concentrations of MG@MIL-100-B composites.

By measuring the peak area ratio of an analyte, the peak area ratio of the corresponding quality control samples of the same concentration was estimated (*n* = 6). The increase in the peak area ratio of these compounds was compared with the area ratio of the same concentration measured in the standard solution of each aqueous solution. Calculation of the matrix effect is presented below:
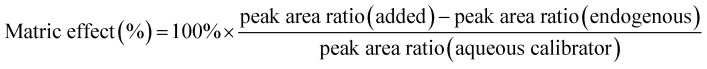


## Conclusions

In this study, novel MG@MIL-100-B nanoparticles were fabricated using the facile MLFC strategy by introducing functionalized mesopores into MOFs. The MG@MIL-100-B composites were employed to extract DA, E and NE from rat plasma successfully. High magnetization of MG@MIL-100-B composites meant that the manipulation complied well with a magnet outside. Furthermore, the MG@MIL-100-B nanoparticles had strong selectivity towards boronic acid groups within the structure and had large adsorption capacity. After enrichment with MG@MIL-100-B sorbents, DA, E and NE in rat plasma were determined by HPLC-MS/MS analysis. The results suggested that the developed MG@MIL-100-B composites were a powerful material to enrich catecholamines in plasma matrix, and that out method serves as a potential tool to quantify trace amounts of catecholamines analytes in complicated matrices. Thus, this approach presents an advanced sample pre-treatment method for clinical study and therapy.

## Conflicts of interest

There are no conflicts to declare.

## Supplementary Material

RA-008-C8RA07356B-s001

## References

[cit1] Rozet E., Morello R., Lecomte F., Martin G. B. (2006). J. Chromatogr. B: Anal. Technol. Biomed. Life Sci..

[cit2] de Jong W. H., de Vries E. G., Wolffenbuttel B. H., Kema I. P. (2010). J. Chromatogr. B: Anal. Technol. Biomed. Life Sci..

[cit3] Li W. D., Scott T. (2000). J. Pharm. Biomed. Anal..

[cit4] Tsunoda M., Aoyama C., Nomura H., Toyoda T. (2010). J. Pharm. Biomed. Anal..

[cit5] Jiang L. W., Chen Y. B., Luo Y. M., Tan Y. M. (2015). J. Sep. Sci..

[cit6] Fujino K., Yoshitake T., Kehr J., Nohta H. (2003). J. Chromatogr. A.

[cit7] Makoto T., Kazuko T., Tomofumi S., Kazuhiro L. (1999). Anal. Biochem..

[cit8] Bicker J., Fortuna A., Alves G., Falcao A. (2013). Anal. Chim. Acta.

[cit9] Zhu K. Y., Fu Q., Leung K. W., Wong Z. C. (2011). J. Chromatogr. B: Anal. Technol. Biomed. Life Sci..

[cit10] Gu Q., Shi X. Z., Yin P. Y., Gao P. (2008). Anal. Chim. Acta.

[cit11] Zhang G. D., Zhang Y. Z., Ji C. J., McDonald T. (2012). J. Chromatogr. B: Anal. Technol. Biomed. Life Sci..

[cit12] Souverain S., Rudaz S., Veuthey J. L. (2004). J. Chromatogr. B: Anal. Technol. Biomed. Life Sci..

[cit13] Huang Z. Z., Lee H. K. (2015). J. Chromatogr. A.

[cit14] Gu Z. Y., Yang C. X., Chang N., Yan X. P. (2012). Acc. Chem. Res..

[cit15] Lee J., Farha O. K., Roberts J., Scheidt K. A. (2009). Chem. Soc. Rev..

[cit16] Joseph D. R., Liu D. M., Lin W. B. (2011). Acc. Chem. Res..

[cit17] Sun N. R., Zhang X. M., Deng C. H. (2015). Nanoscale.

[cit18] Frans B., Gooltzen A., Loes E. (1993). Clin. Chem..

[cit19] Talwar D., Williamson C., McLaughlin A., Gill A. (2002). J. Chromatogr. B: Anal. Technol. Biomed. Life Sci..

[cit20] Lin Z. A., Zheng J. N., Lin F., Zhang L. (2011). J. Mater. Chem..

[cit21] Zhang H., Yao G., Lu H., Deng C. (2011). Chin. J. Chem..

[cit22] Park J., Wang Z. U., Sun L. B., Chen Y. P. (2012). J. Am. Chem. Soc..

[cit23] Burtch N. C., Jasuja H., Walton K. S. (2014). Chem. Rev..

[cit24] Zhu X. Y., Gu J. L., Zhu J. Y., Li Y. S. (2015). Adv. Funct. Mater..

[cit25] Sun X. N., Liu X. D., Feng J. N., Li Y. (2015). Anal. Chim. Acta.

[cit26] Tong M. M., Liu D. H., Yang Q. Y., Devautour-Vinot S. (2013). J. Mater. Chem. A.

[cit27] Chen Y. J., Xiong Z. C., Peng L., Gan Y. Y. (2015). ACS Appl. Mater. Interfaces.

[cit28] Kumarathasan P., Vincent R. (2003). J. Chromatogr. A.

[cit29] Sastre E., Nicolay A., Bruguerolle B., Portugal H. (2004). J. Chromatogr. B: Anal. Technol. Biomed. Life Sci..

[cit30] Machida M., Sakaguchi A., Kamada S., Fujimoto T. (2006). J. Chromatogr. B: Anal. Technol. Biomed. Life Sci..

[cit31] Raggi M. A., Sabbioni C., Casamenti G., Gerra G. (1999). J. Chromatogr. B: Biomed. Sci. Appl..

[cit32] Sabbioni C., Saracino M. A., Mandrioli R., Pinzauti S. (2004). J. Chromatogr. A.

