# Supplementary material for: Facile synthesis of boronic acid-functionalized magnetic metal–organic frameworks for selective extraction and quantification of catecholamines in rat plasma
Source: RSC Adv. 2018 Dec 17;8(73):41976–85. doi: 10.1039/c8ra07356b (PMC9092088; doi:10.1039/c8ra07356b)
Supplement: RA-008-C8RA07356B-s001 [file RA-008-C8RA07356B-s001.pdf]

Electronic Supplementary Material (ESI) for RSC Advances.
This journal is © The Royal Society of Chemistry 2018

Supporting information:

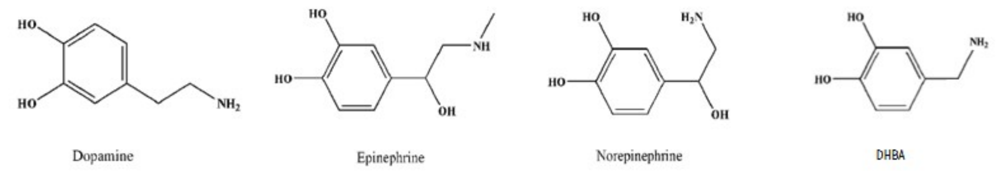

Fig. S1. The chemical structures of the dopamine (DA), epinephrine (E), norepinephrine (NE) and 3,4-dihydroxybenzylamine (DHBA)

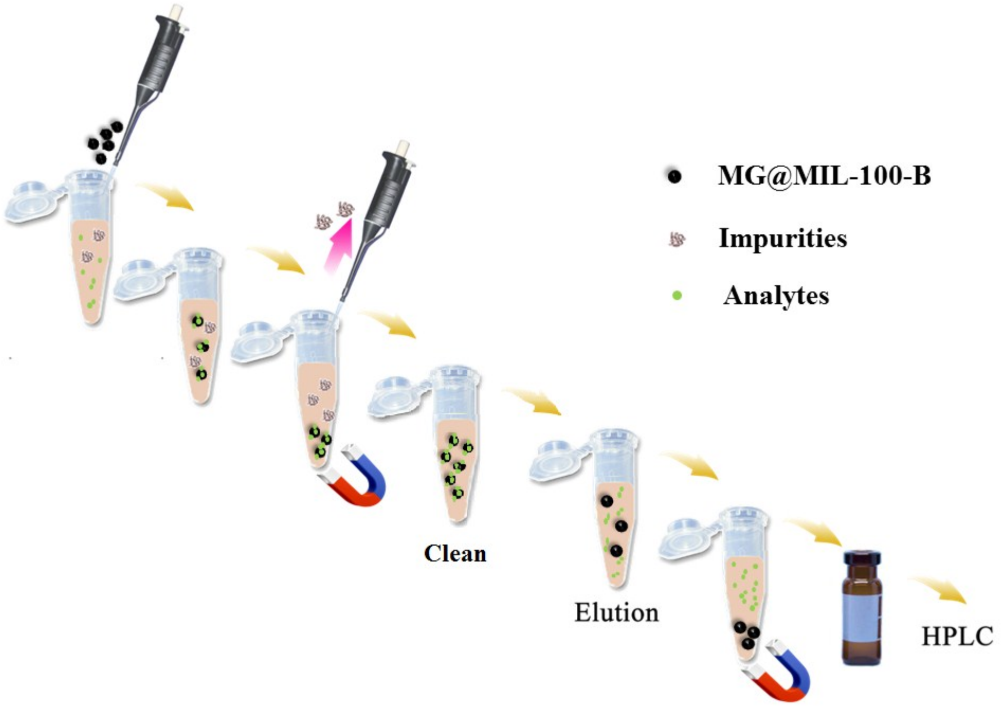

Fig. S2. Systematic route of the extraction procedure
